# Reduced loss aversion in pathological gambling and alcohol dependence is associated with differential alterations in amygdala and prefrontal functioning

**DOI:** 10.1038/s41598-017-16433-y

**Published:** 2017-11-24

**Authors:** Alexander Genauck, Saskia Quester, Torsten Wüstenberg, Chantal Mörsen, Andreas Heinz, Nina Romanczuk-Seiferth

**Affiliations:** 1 Department of Psychiatry and Psychotherapy, Charité – Universitätsmedizin Berlin, corporate member of Freie Universität Berlin, Humboldt-Universität zu Berlin, and Berlin Institute of Health, Berlin, Germany; 2 Bernstein Center for Computational Neuroscience Berlin, Berlin, Germany; 3 Berlin School of Mind and Brain, Humboldt-Universität zu Berlin, Berlin, Germany

## Abstract

Diagnostic criteria for pathological gambling and alcohol dependence (AD) include repeated addictive behavior despite severe negative consequences. However, the concept of loss aversion (LA) as a facet of value-based decision making has not yet been used to directly compare these disorders. We hypothesized reduced LA in pathological gamblers (PG) and AD patients, correlation of LA with disorder severity, and reduced loss-related modulation of brain activity. 19 PG subjects, 15 AD patients and 17 healthy controls (HC) engaged in a LA task in a functional magnetic resonance imaging setting. Imaging analyses focused on neural gain and loss sensitivity in the meso-cortico-limbic network of the brain. Both PG and AD subjects showed reduced LA. AD subjects showed altered loss-related modulation of activity in lateral prefrontal regions. PG subjects showed indication of altered amygdala-prefrontal functional connectivity. Although we observed reduced LA in both a behavioral addiction and a substance-related disorder our neural findings might challenge the notion of complete neuro-behavioral congruence of substance-use disorders and behavioral addictions.

## Introduction

Value-based decisions are ubiquitous in every-day life. They can be anything from short-term and mundane (tea or coffee) to long-term and life changing (law or medical school). In all of these decisions we need to incorporate magnitude, delay and probability of possible rewards and losses to compute subjective values of the available options^1^. Several psychiatric disorders have been linked to altered neurobehavioral processes of value-based decision-making^2–5^. Pathological gambling (PG) and alcohol dependence (AD) have been classified as addictive disorders alongside each other in the DSM-5 because they show similar neurobehavioral patterns and impairments when performing value-based decision-making tasks and because they show similar clinical symptoms (e.g. craving, tolerance, loss of control)^6–9^. Diagnostic criteria of PG and AD also overlap when it comes to the core features of both disorders. These include reduced aversion against negative consequences of the addictive behavior. Accordingly, loss aversion (LA), a form of magnitude discounting in value-based decision-making, might be affected in both PG and AD. However, to our knowledge, LA has not yet been concurrently investigated and directly compared in these disorders.

LA is the tendency to be more sensitive to the magnitude of possible losses than possible gains when facing mixed gambles^10^. In the case of a mixed gamble having exactly one possible gain outcome with probability 0.5 and one possible loss outcome with probability 0.5 (e.g. a coin toss gamble), healthy subjects usually need to be offered a possible gain which is at least double the size of the possible loss before they agree to gamble^11^.

Reduced LA in PG subjects has been observed before^12–14^. Yet, our study is the first to investigate the neural basis of differences in LA between PG, AD and HC subjects by investigating differences in behavioral and neural sensitivity to possible gains and losses during the decision-making process. Further, we are not aware of any studies investigating LA in AD subjects. However, there have been studies in other substance-use-disorder (SUD) samples (e.g. cocaine and cannabis dependent subjects) which found mostly reduced LA^15–19]^. Yet, these studies have not reported which differences in behavioral and neural gain and loss sensitivity were the basis for differences in LA. In the current study, we expect reduced LA in both PG and AD. This decrease in LA may be due to decrease in behavioral loss sensitivity and/or increase in behavioral gain sensitivity. We further expect that different levels of LA are correlated with different levels of PG and AD symptom severity. This is because other facets of value-based decision-making, namely delay and probability discounting, have been found correlated with PG and AD symptom severity^20–22^.

LA differences have so far been mostly attributed to differences in neural loss sensitivity in cortical and limbic areas^23–26^, which we expect to see as well. In that vein, it has been suggested that possible losses produce a cost signal in dorso-lateral-prefrontal cortex (DLPFC) enhancing the representation of loss values in orbitofrontal-cortex^27^. In line with this, the DLPFC has been implicated as necessary for avoiding risky choices^28,29^. In healthy subjects, DLPFC and the ventro-medial prefrontal cortex (VMPFC) have been found correlated with a cost-benefit signal in an fMRI study^30^. The two areas seemed to be most active if gains were subjectively larger than gains and least active when losses were subjectively bigger than gains. According to this, we hypothesize: With increasing losses HC subjects should show stronger *decrease* in DLPFC activity than both PG and AD subjects.

LA studies in healthy subjects have observed that apart from DLPFC a whole network of brain areas is increasing activity with gains and decreasing activity with losses^25,31^. Studies on reward anticipation in PG and AD additionally suggest altered striatal functioning for explaining reduced LA in PG and AD subjects^32–37^. Studies on factors influencing LA, such as focal brain damage^23^, sleep deprivation^24^, emotion regulation^38^ and modulation^39^, as well as studies on cognitive control^40,41^ imply additional brain areas for explaining inter-individual differences in LA. We thus test for altered Blood-Oxygenation-Level-Dependent (BOLD) reactivity in PG and AD subjects with respect to both gains and losses in a LA network of interest (NOI) encompassing the regions of interest (ROIs) DLPFC, ventro-lateral prefrontal cortex (VLPFC), orbito-frontal cortex (OFC), amygdala, insula, VMPFC, striatum, midbrain, and dorsal raphe nucleus (DRN).

## Materials and Methods

### Loss aversion task

**Figure 1:**
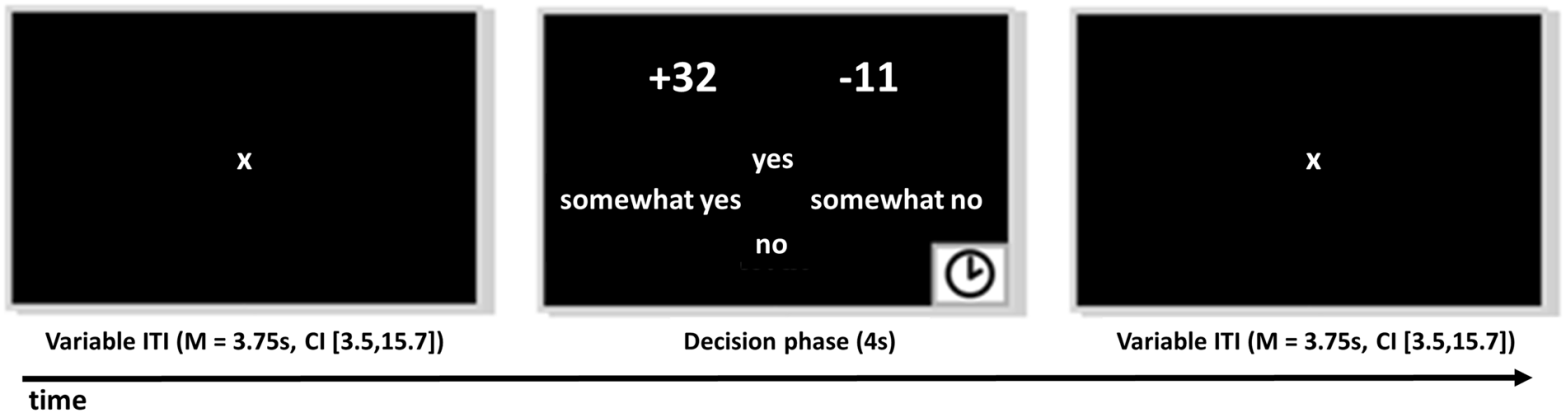
The loss aversion task^31^. One trial is depicted. Subjects first saw a fixation cross with variable inter-trial-interval (ITI). Subjects then saw a gamble involving a possible gain and a possible loss. Position of gain and loss was counterbalanced (left/right). Gain was indicated by a ‘+’ sign and loss by a ‘−’ sign. Subjects had 4s to make a choice between four levels of acceptance (English levels here only used for illustration; in German “ja”, “eher ja”, “eher nein”, “nein” were used). Directly after decision, the ITI started. If subjects failed to make a decision within 4s, ITI started and trial was counted as missing. M… mean; CI… 95% Confidence Interval.

We used an established task to measure LA^31^. Subjects were each told that they had 20€ for wagering. On every trial, subjects were presented with a mixed gamble, involving a possible gain and a possible loss with probability P = 0.5 each. Subjects were asked to indicate willingness to accept the gamble (Fig. 1). Gambles were created by randomly drawing with replacement from a matrix with possible gambles consisting of 12 levels of gains (14, 16, …, 36) and 12 levels of losses (−7, −8, …, −18). This matrix is apt to elicit LA in healthy subjects^31^. 144 gambles were presented. We informed subjects that after the scanning session five of their gamble decisions with ratings of “rather yes” or “yes” would be randomly chosen and played for real money.

### Sample

Subjects had to be male, right-handed, and eligible for fMRI scanning. AD patients were diagnosed by a psychiatrist according to ICD-10 and DSM-IV criteria. The psychiatrist confirmed that AD patients did not fulfill the criteria for PG. AD patients were recruited from an in-patient detoxification ward. AD detoxification took place on average 42 days before scanning (CI_boot95%_ = [28, 60]). PG subjects were recruited via internet advertisement and notices in casinos. PG subjects were diagnosed using the German short questionnaire for gambling behavior (KFG)^42^ by a trained psychologist (SQ). Otherwise, any known history of a neurological disorder or a current psychological disorder (except alcohol abuse for the AD group and tobacco dependence for all three groups) as assessed by the screening of the Structured Clinical Interview for DSM-IV Axis I Disorders (SCID-I) lead to exclusion from the study. PG subjects additionally completed the Yale Brown Obsessive Compulsive Scale adapted for PG (PGYBOCS) to measure severity of gambling behavior over a recent time interval^43^, as well as the Gambling Symptom Assessment Scale (G-SAS)^44^ as another symptom severity scale. AD patients completed the Alcohol Dependence Scale (ADS) as a severity measure^45^ and the Obsessive Compulsive Drinking Scale as a craving measure (OCDS)^46^. All subjects completed the Gamblers’ Beliefs Questionnaire (GBQ) asking for belief in gambling persistence to achieve wins and for gambling illusions^47^. Symptom severity measures were chosen to check if the LA score relates to clinical symptom severity^48^. There were 6 subject dropouts (1 misunderstanding of task instructions, 5 technical error). Within the group of PG subjects, 17 indicated slot machines as their primary gamble and 2 indicated sports betting. There was one PG subject that had a history of diagnosed alcohol dependency but no current alcohol dependency. For further information on administered questionnaires, see Supplementary Information. The final sample consisted of 19 PG, 15 AD and 17 HC subjects (Table 1).

**Table 1:** Differences between groups in demographic and clinical data.

**Variable**	**Group**	**Test Statistics**											
	**HC**			**PG**			**AD**			**MAIN**	**HC**	**HC**	**AD**
	**M**	**SD**	**NA**	**M**	**SD**	**NA**	**M**	**SD**	**NA**	**EFFECT**	**<>**	**<>**	**<>**
										**GROUP**	**AD**	**PG**	**PG**
										**p**	**p**	**p**	**p**
**Age**	38.8	11.5	0	32.9	10	0	45.4	10.2	0	**<0.01** ^**a**^	**0.08** ^**a**^	**0.10** ^**a**^	**<0.01** ^**a**^
**Cigarettes per day**	5.9	7.8	0	6.4	8.4	0	13.6	10.1	0	0.06^b^	—	—	—
**Intelligence**	18.6	4.6	0	17.9	3.4	0	17.3	4.6	0	0.55^b^	—	—	—
**Years of education**	16.3	3.4	0	14.1	3.3	0	16.6	4.7	0	0.07^b^	—	—	—
**Years in school**	11.3	1.6	0	11	1.7	0	11.3	1.9	0	0.78^b^	—	—	—
**Impulsivity I**	66	9	0	82	13	0	75	8	0	**<0.01** ^**a**^	**0.03** ^**a**^	**<0.01** ^**a**^	0.09^a^
**Impulsivity II**	59	8.8	0	73	11.1	0	66	7.3	0	**<0.01** ^**a**^	**0.03** ^**a**^	**<0.01** ^**a**^	0.07^a^
**Depression**	3.8	4.8	1	12.5	9	0	12.7	7.9	0	**<0.01** ^**b**^	**<0.01** ^**b**^	**<0.01** ^**b**^	0.66^b^
**Debt (yes/no)**	5/11	—	1	14/4	—	1	9/4	—	2	**0.02** ^**c**^	0.07^c^	0.01^c^	0.69^c^
**Debt (euros)**	0^1)^	—	6	3000^1)^	—	4	4500^1)^	—	5	**<0.01** ^**b**^	**<0.01** ^**b**^	**<0.01** ^**b**^	0.36^b^
**Personal income** ^2^	985	305	0	795	478	1	1033	863	0	0.36^b^	—	—	—
**Handedness**	81.9	39.3	0	65.3	66.6	0	76	51	0	0.91^b^	—	—	—
**PG severity I**	2.1	2.8	0	33.2	9.9	0	1.73	6.7	1	**<0.01** ^**b**^	**0.04** ^**b**^	**<0.01** ^**b**^	**<0.01** ^**b**^
**PG severity II**	2.2	2.9	0	21.2	9.4	0	0.37	1.29	0	**<0.01** ^**b**^	**0.02** ^**b**^	**<0.01** ^**b**^	**<0.01** ^**b**^
**Gamblers beliefs**	1.6	0.8	0	2.4	0.5	0	1.4	0.6	2	**<0.01** ^**b**^	0.41^b^	**<0.01** ^**b**^	**<0.01** ^**b**^
**PG illusions**	1.6	0.8	0	2.4	0.6	0	1.4	0.6	2	**<0.01** ^**b**^	0.50^b^	**<0.01** ^**b**^	**<0.01** ^**b**^
**PG persistence**	1.5	0.8	0	2.4	0.6	0	1.3	0.8	2	**<0.01** ^**b**^	0.50^b^	**<0.01** ^**b**^	**<0.01** ^**b**^
**PG craving**	10.8	1.6	1	24.6	6.8	0	10.9	2.6	0	**<0.01** ^**b**^	0.68^b^	**<0.01** ^**b**^	**<0.01** ^**b**^
**AD craving**	0.6	1.2	1	1.3	2.2	0	5.3	4.5	0	**<0.01** ^**b**^	**<0.01** ^**b**^	<0.26^b^	**<0.01** ^**b**^
**AD severity**	—	—	—	—	—	—	19	6.7	0	—	—	—	—

PG, pathological gambling; AD, alcohol dependence; NA, number of missing values due to technical error or refusal by subject to answer, replaced by median of respective group, except in Debt variables; M, mean; SD, standard deviation; Intelligence measured by Wechsler Adult Intelligence Scale matrices test; Depression measured by Beck’s Depression Inventory II; Impulsivity I measured by Barrat Impulsiveness Scale 10; Impulsivity II measured by BIS-11, i.e. BIS-10 dropping items 19,26,27,29; Handedness measured by Edinburgh Handedness Inventory; PG severity I measured by Kurzfragebogen Spielsucht (short gambling questionnaire); PG severity II measured by Gambling Symptom Assessment Scale (G-SAS); Gamblers beliefs measured by Gamblers Beliefs Questionnaire (GBQ); Illusions measured by illusions subscale of GBQ; persistence measured by GBQ persistence subscale; PG craving measured by Yale-Brown Obsessive Compulsive Scale for Pathological Gambling; AD craving measured by Obsessive Compulsive Drinking Scale; AD severity measured by Alcohol Dependence Scale; see Supplementary for references of questionnaires; ^a^p-value of general linear model (GLM) with group as predictor, or p-value of respective contrast; bold: significant difference; ^b^p-value of Kruskal-Wallis Rank Sum Test with group as predictor or for respective contrast; ^c^Fisher’s Exact Test for Count Data; ^1)^median; ^2)^if subject refused answer, household income divided by the number of persons living in the household was used; in case of GLM’s, assumption of normal distribution of residuals was not rejected according to Shapiro-Wilk Test. Assumption of equality of variances between groups was tested using Bartlett Test of Homogeneity of Variances; if either failed, Kruskal-Wallis Rank Sum Test test was used.

### Procedure and data acquisition

All subjects gave written informed consent. The study was conducted in accordance with the latest version of the Declaration of Helsinki and approved by the Ethics Committee of the Charité - Universitätsmedizin Berlin. All subjects underwent T2*-sensitive Gradient-Echo Echo-Planar scanning during task completion and a T1-weighted structural scan. See Supplementary Information for further description of scanning sequences used.

### Analysis of behavioral data

Gain and loss variables were down-sampled, yielding a 4-by-4 gamble matrix. Losses were used as absolute values. Gains and losses were centralized. Choices of subjects were dichotomized to “yes” and “no” and entered into a mixed effects logistic regression in the framework of the Generalized Linear Model using the lme4 package in R^49^. We chose mixed effects modeling because it yields less outlier-prone subject parameters. Several LA models were considered and the following one was chosen, because of good fit and because it allowed us to disentangle general acceptance rate, behavioral gain and loss sensitivity (see Supplementary Methods). Predictors were gain, loss, and group membership as fixed effects sources. Subjects were included as a source of random effects on all fixed effects, including the intercept. The group specific fixed effect LA parameter λ was then defined as:

1 $$\lambda =-{\beta }_{loss}/{\beta }_{gain}$$

Here, ${\beta }_{loss}$ and ${\beta }_{gain}$ are the regression weights for the group specific fixed effects of behavioral gain and loss sensitivity, respectively. Subjective utilities of gains and losses were assumed to increase linearly with increasing gains and losses^11^. All statistical analyses of the behavioral data were conducted using R (version: 3.2.2)^50^.

To test for an effect of group the LA model with group was compared with the LA model without group. A significant effect of group was assumed if the chi-square difference test was significant (p < 0.05) and if the Aikaike Information Criterion (AIC) value of the model with group was lower than of the LA model without group as predictor. Parametrically bootstrapped p-values (p_boot_) for post-hoc contrasts for *λ*, *β*_*loss*_ and *β*_*gain*_ (HC > PG, HC > AD, PG > AD, PG < AD) were obtained by running 1000 simulations of the model without group as predictor. To test whether effects were robust against adjusting for group differences in covariates of no interest, the analysis procedure was repeated with age as an additional predictor^51^, where group and age were allowed to modulate the intercept, as well as *β*_*loss*_ and *β*_*gain*_. Symptom severity scores (AD: ADS, OCDS; PG: KFG, PGYBOCS, G-SAS, GBQ) were Pearson correlated with log(*λ*) in PG and AD groups. In each group bootstrapped p-values were computed for each correlation coefficient and FDR corrected for multiple tests (2 in AD and 4 in PG) at an alpha level of 0.05^52^.

### Analysis of imaging data

Imaging analyses were performed in SPM12 running on Matlab (R2014a). Please see Supplementary Methods for description of preprocessing of MRI data. The preprocessed fMRI single-subject data was modeled using a boxcar function denoting times of gamble presentation (task-on regressor) and three linearly scaled task-on regressors (gain and loss parallel to behavioral analysis plus Euclidean distance based on aggregated gamble matrix^31^). Note that this model is completely in parallel with the behavioral model – only the dependent variable differs. In the behavioral model it is choice, in the neural model it is BOLD activity. The regressors were convolved with the canonical hemodynamic response function, downsampled to match the number of EPI scans and entered into a GLM. For further details on the single-subject model, please see Supplementary Methods.

Contrast images for gain (“neural gain sensitivity”) and loss (“neural loss sensitivity”) of all participants were subjected to two separate one-way ANOVAs with group as predictor and assumption of non-equal variance between groups. Main effect (ME) group F-Test images were computed for gain and loss and thresholded at p < 0.05, minimum cluster extent k = 10. Group main effect F-test maps were then corrected for family-wise error (FWE) at peak level using small volume correction (SVC) with our network of interest (NOI, see Supplementary and online.nii file) as small volume. Note, that since the group comparison hypotheses were the same in all of the regions within the NOI it is the most stringent approach to perform *one* SVC for the whole NOI in the neural gain and neural loss sensitivity analysis, respectively. Then all possible one-sided post-hoc T-test images to compare HC, PG, AD were computed and peak-level FWE corrected using the NOI. Significant peak voxels from post-hoc T-tests were only considered if the FWE corrected F-Test before yielded the respective voxel also as significant.

Since gray matter density (GMD) in both AD and PG has been observed different from HC^53,54^, and since there were significant group differences in a covariate of no interest, all found group differences in post-hoc T-test at voxels with significant SVC correctable F-Test were checked for stability by rerunning the analyses with local GMD and age using robust Biological Parametric Mapping (rBPM) with Tukey’s biweight error function using the BPMe toolbox^51,55,56^ (small shifts of peak voxels within the respective ROI were allowed) (see Supplementary Methods).

### Exploratory analyses

To further explore the neural basis *of group differences in behavioral loss aversion* we tested for functional connectivity group differences in our NOI. We computed functional connectivity maps using generalized psycho-physiological interaction analysis (gPPI)^57,58^ using seed regions according to the affective neuroscience of decision through reward-based evaluation of alternatives (ANDREA) model^27^ and the connectivity model by Basten *et al*.^30^. Obtained gain-related and loss-related functional connectivity parameters reflected how correlation of the signal between the signal of the respective seed region and all other voxels was changing with respect to rising gains, or losses, respectively. Connectivity maps were submitted to all possible one-sided T-tests comparing HC, PG, AD. Significant AD > PG or AD < PG results were only reported if in the same connectivity and peak voxel PG and AD also significantly differed from HC. For FWE correction we used target anatomical ROIs, as implied by the connectivity models (Table 2).

**Table 2:** Exploratory functional connectivity analyses.

**seed**	**target ROIs**	**contrast of interest**	**number of one-sided T-tests** ^3)^
*ANDREA* ^27^ *network model*			
VS	ACC	[gain, loss]	12
DLPFC^1)^	ACC	[loss]	12 (per BA)
dorsal raphe nucleus (DRN)	DLPFC^1)^, amygdala	[loss]	60
VTA/midbrain	amygdala	[gain]	12
ACC	DLPFC^1)^, amygdala	[gain, loss], [loss]	60
amygdala	OFC^2)^	[gain, loss]	48
OFC	DRN, VTA	[loss], [gain]	12 (per OFC sub area)
*Basten* ^30^ *connectivity model*			
VS	VMPFC	[gain]	12
amygdala	VMPFC	[loss]	12

^1^BA 8, 9, 10, 46 within MFG considered separately.

^2^Anterior, lateral, medial, posterior orbital gyrus considered separately.

^3^All T-tests done separately for left/right of seed and target ipsilaterally and contralaterally.

Connectivity maps were computed for every left and right seed region separately, except for right VS because of signal loss (23 maps). All target ROI FWE correction was done for left and right separately. Found group differences in functional connectivity were checked for stability against adjusting for age using ancova analysis in SPM. Only results are reported which survived adjustment for age.

To further explore the neural basis of the *relationship of symptom severity with behavioral LA within groups*, we correlated symptom severity (**AD**: ADS, OCDS; **PG**: KFG, PGYBOCS, G-SAS, GBQ), with neural gain sensitivity, neural loss sensitivity, as well as with neural loss aversion maps^25,31^. We used our NOI for SVC on the ensuing one-sample T-test maps. Neural loss aversion (nLA) maps were computed by subtracting in every subject the neural gain sensitivity image from the negative neural loss sensitivity image (-loss - gain; since losses were entered as absolute values in single-subject model)^25,31^.

### Availability of materials and data

The datasets generated during and analyzed during the current study are available from the corresponding author on reasonable request. FMRI T-maps are available at https://neurovault.org/collections/3163/.

## Results

### Behavior

**Figure 2:**
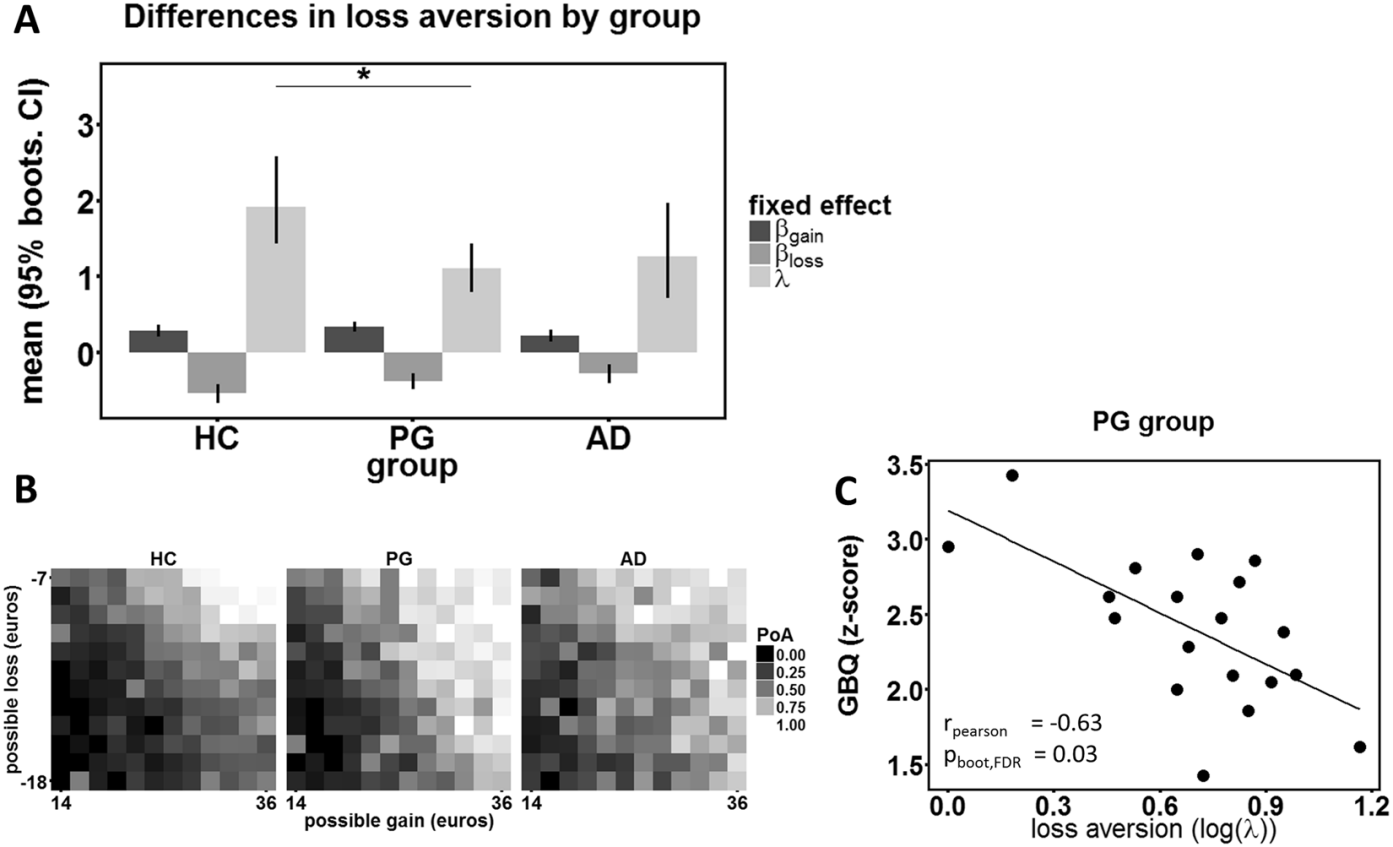
Behavioral results by group. (**A**) Pathological gamblers (PG) and alcohol dependent (AD) patients show similarly reduced loss aversion (LA, 𝜆). AD and PG subjects show significantly reduced behavioral loss sensitivity (β_loss_). (**B**) Differences in LA as seen in probability of gamble acceptance (PoA) maps. PoA was calculated within each subject and for each gamble cell based on the frequency of gamble acceptance divided by number of gamble presentations. Then a mean PoA map was calculated for each group. Light grey indicates high PoA and dark grey indicates low PoA. Note that AD and PG subjects change their acceptance rate less strongly with respect to changing magnitude of losses, compared to healthy controls (HC), i.e. show reduced behavioral loss sensitivity. (**C**) Correlation of behavioral loss aversion with GBQ (Gambler’s Beliefs Questionnaire) in PG subjects. In GBQ high values code for high cognitive distortions.

Inclusion of group into the behavioral model was significant, p(ΔChi^2^) = 0.002, ΔAIC = 9.1. The HC group showed a fixed effect of *λ* of 1.89, the AD group a *λ* of 1.23 and the PG group a *λ* of 1.09, (Fig. 2). HC’s LA was greater than that of both PG and AD (HC > PG, p_boot_ = 0.014; HC > AD, p_boot_ = 0.042). PG and AD did not differ in LA (PG > AD, p_boot_ = 0.636). LA results stayed the same with age as covariate in the model. AD and PG patients showed a reduction in *β*_*loss*_ compared to HC (p_boot_ = 0.009; p_boot_ = 0.019) (Fig. 2), robust against adjusting for age. Both groups did not differ from HC nor between each other in *β*_*gain*_. HC subjects did not change their reaction time with gains or losses. PG and AD subjects did so, with increasing gains decreasing their reaction time and increasing losses increasing their reaction times (see Supplementary Information, Fig. S2).

No AD severity measure correlated with LA in AD subjects. In PG subjects log(*λ*) correlated significantly only with the GBQ score, r = −0.63, p_boot_,_FDR_ = 0.03 (Fig. 2C). In exploratory analyses we found that this was driven by both the GBQ illusions subscale (r = −0.72, p_boot_ = 0.004) and by the GBQ persistence subscale (r = −0.47, p_boot_ = 0.03).

### Brain response

**Figure 3:**
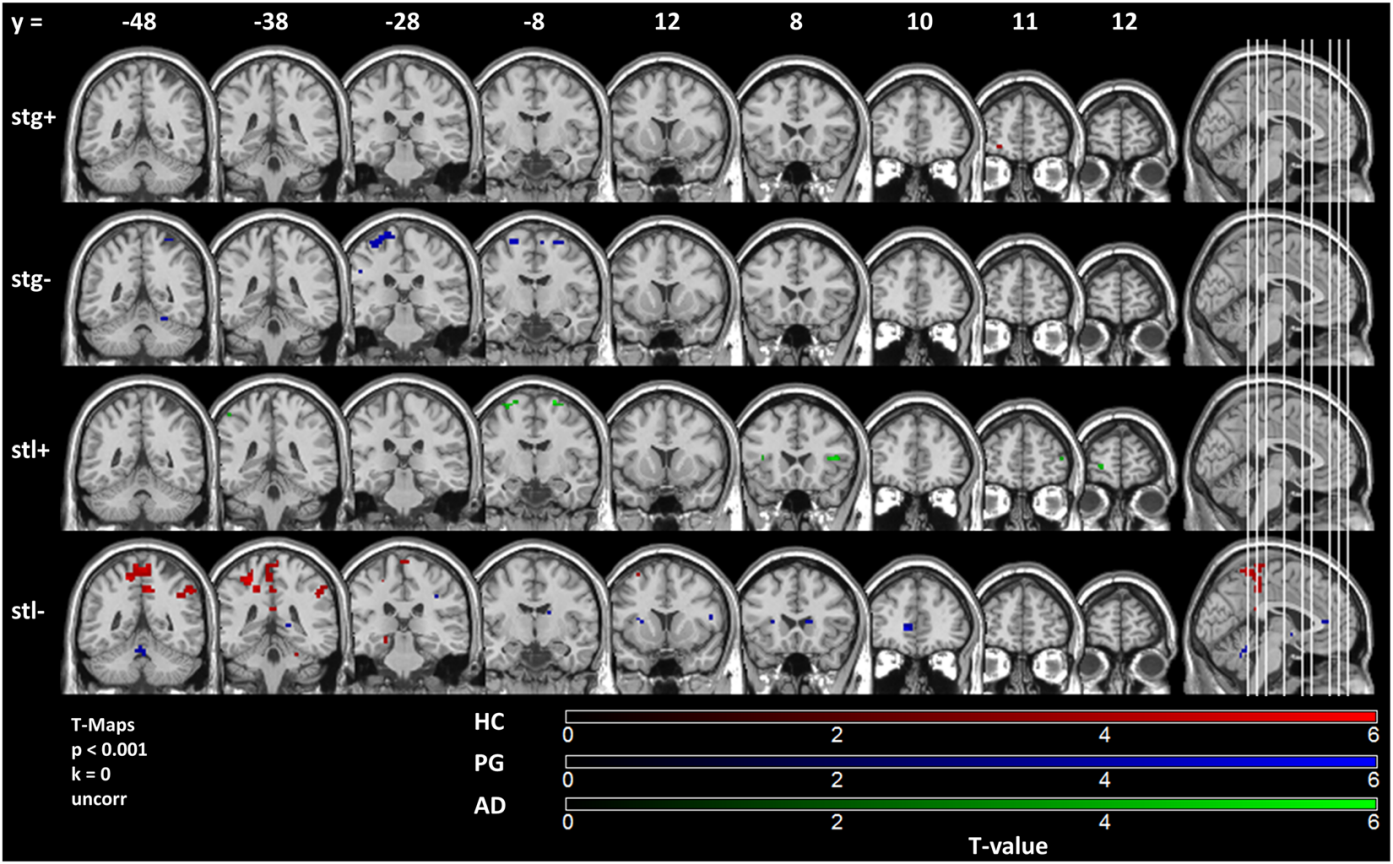
Effects of task in HC, PG, AD. T-Test maps of correlation of BOLD activity with gain or absolute value of loss superimposed on canonical SPM12 T1-image, thresholded at p < 0.001, uncorrected, extent threshold k = 0. HC: healthy controls; PG: pathological gamblers; AD: alcohol dependent patients; stg +: positive correlation of gain with BOLD activity; stg −: negative correlation of gain with BOLD activity; stl +: positive correlation of absolute loss with BOLD activity; stl −: negative correlation of absolute loss with BOLD activity.

Neither whole brain nor NOI SVC correction yielded significant peak voxels in neural gain sensitivity or neural loss sensitivity T-maps in any of the groups. We also explored T-maps at p < 0.001, cluster extent threshold k = 0. In HC subjects, with rising gains, BOLD activity increased in left middle frontal gyrus/left anterior orbital gyrus, medial superior frontal gyrus, left caudate. Decrease in BOLD activity with rising gains was non-existent in HC subjects. In HC subjects, BOLD activity decreased with rising losses in left cerebellum exterior, left superior parietal lobule, left and medial postcentral gyrus, bilateral precuneus, left thalamus/left parahippocampal gyrus/left hippocampus, right supamarginal/angular gyrus, right middle frontal gyrus. HC subjects did not show any BOLD increase with rising losses. PG subjects showed increasing activity in right superior frontal gyrus with rising losses and decreasing activity with rising losses in bilateral anterior cingulum, right caudate, left putamen, left insula, bilateral inferior frontal operculum, left rolandic operculum, bilateral diencephalon, right pre- and postcentral gyrus, right supramarginal gyrus, medial cerebellum. PG subjects showed activity increase in left hippocampus with rising gains. PG subjects showed activity decrease with rising gains in left superior parietal lobe, right precentral gyrus, left/right superior frontal gyrus, right supplementary cortex, left precentral/supramarginal gyrus, occipital gyrus, cerebellum. AD subjects showed increasing BOLD activity in response to rising losses in right middle frontal and bilateral superior frontal gyrus, as well as in bilateral frontal operculum, and bilateral precentral gyrus. AD subjects did not show decreasing activity with rising losses. AD subjects showed neither increasing nor decreasing activity in any region with rising gains (see selection of slices in Fig. 3).

Comparing the groups, main effect of group (ME) F-Test for the neural sensitivity to loss maps yielded after NOI SVC correction two significant peak voxels: [48,49,5], DLPFC (middle frontal gyrus, BA46), p_FWE_ = 0.012, and [59,14,16] (VLPFC, opercular part of the inferior frontal gyrus, BA44/BA45), p_FWE_ = 0.026.

**Figure 4:**
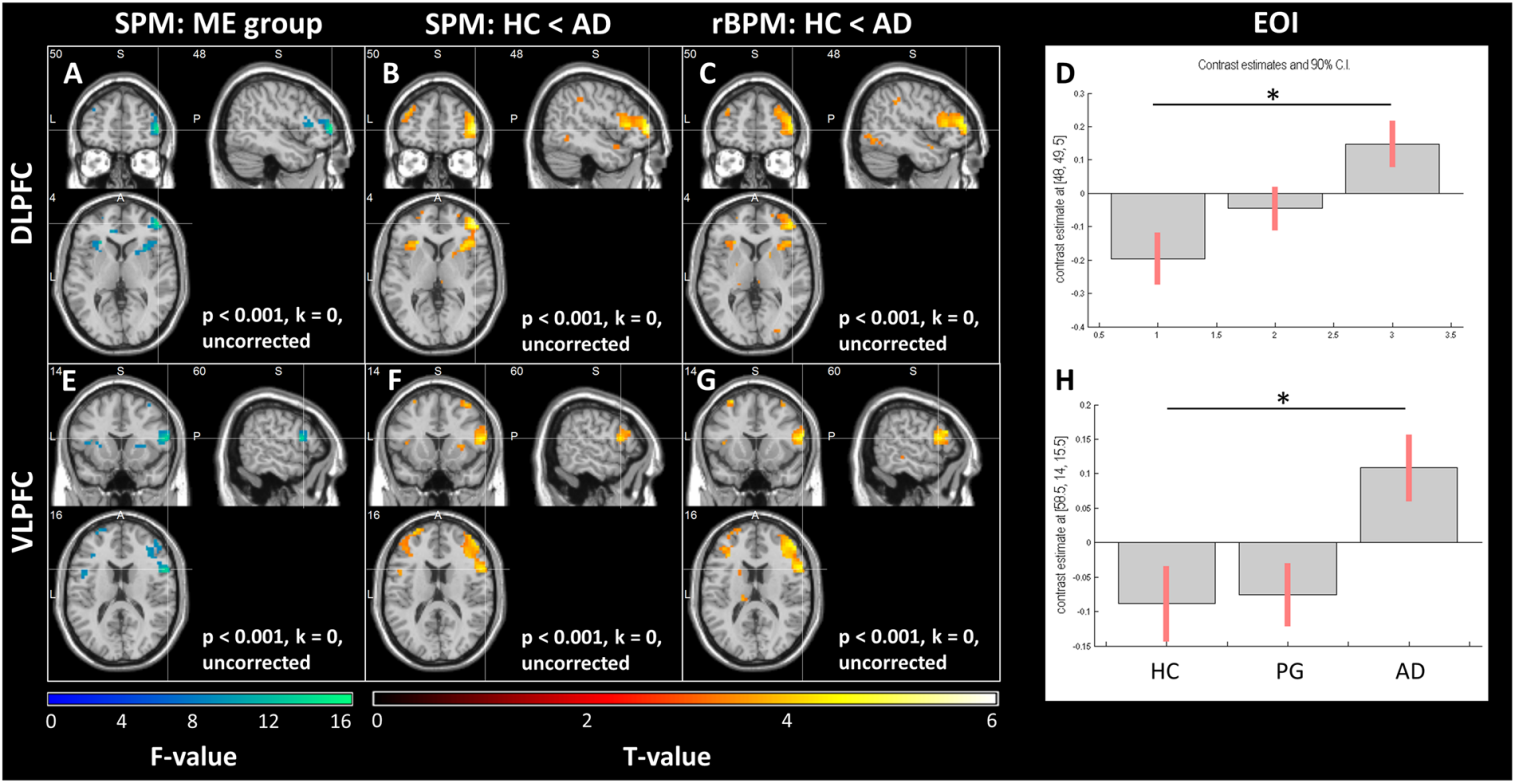
Neural loss sensitivity group differences, HC < AD. Heatmaps show significant activation at p < 0.001, k = 0, uncorrected. (**A,E)** Main effect (ME) of group F-map. (**B**,**F**) T-map for contrast HC < AD for the neural loss sensitivity contrast. (**C**,**G)** rBPM analysis. I.e. HC < AD T-Test adjusted for age and local gray matter density. (**D**,**H)** Unadjusted means of neural loss sensitivity per group (effect of interest, EOI). For illustration coherence, panels A-D are focused on the significant peak voxel of the main effect of group at [48,49,5] in right DLPFC (BA46 in right middle frontal gyrus) and (**E**–**H**) at [59,14,16] in VLPFC (BA44/BA45 in inferior frontal gyrus).

Post-hoc T-Tests revealed: 1) a significant group comparison stable against adjusting for age and local gray matter density (using rBPM) for the **HC < AD** contrast at [48, 49, 5]. With rising losses, HC subjects showed in right DLPFC a stronger reduction of activity than AD patients, p_FWE_ = 0.001, t = 5.47, p < 0.001, p_FWE(rBPM)_ = 0.040 (in rBPM slight shift of peak voxel to [48, 46, 12] and [52, 42, 16], both DLPFC, BA46), k = 713 (Fig. 4). 2) a significant group comparison stable against adjusting for age and local gray matter density (using rBPM) for the **HC** < **AD** contrast at [59, 14, 16]. With rising losses, HC subjects showed in right VLPFC a stronger reduction of activity than AD patients, p_FWE_ = 0.025, t = 4.53, p < 0.001 (slight shift to [55, 14, 12], VLPFC, BA44), p_FWE(rBPM)_ = 0.021 (slight shift to [62,14,19], VLPFC, BA45) (Fig. 4). Post-hoc T-Tests comparing HC and PG, as well as PG and AD, yielded no correctable results at points of significant ME group. Whole brain FWE correction of the ME group F-map for neural loss sensitivity yielded no significant voxels (trend at [48,49,5], p_FWE_ = 0.058). There were no significant group differences in neural gain sensitivity, neither when using our NOI, nor when using the whole brain FWE correction.

### Results of exploratory analyses

#### Functional connectivity

**PG > HC:** We found PG subjects showing a stronger gain-related functional connectivity from left amygdala to left posterior OFC, [−29 14–20], p_FWE_ = 0.017, k = 12 (Fig. S2-A), meaning that with rising gains correlation of the BOLD signal between amygdala and OFC increased in PG subjects more strongly than in HC subjects. PG subjects also showed this from right amygdala to left post. OFC, [−29 18–20], p_FWE_ = 0.004, k = 35, (Fig. S4-B). **HC > PG:** In PG subjects we found that loss-related functional connectivity from left amygdala to VMPFC is weaker in PG subjects than in HC subjects, [−1 56–6], p_FWE_ = 0.024, k = 44, (Fig. S4-C), meaning that with rising losses correlation of the BOLD signal between amygdala and VMPFC increased in HC subjects more strongly than in PG subjects. The same was true for functional connectivity between left posterior OFC and DRN/brain stem, [−1, −32, −13], p_FWE_ = 0.018 (Fig. S4-D).

#### Correlations of neural LA parameters with symptom severity scores

There were no correlations of neural sensitivity to gain/neural sensitivity to loss/nLA with symptom severity scores within PG nor AD using our NOI for SVC.

## Discussion

Impaired value-based decision-making is a hallmark of both substance-related disorders and pathological gambling^59,60^. We have further probed the neuro-behavioral factors associated with impaired decision making in both PG and AD focusing on group differences in LA. We observed that both PG and AD subjects show reduced LA compared to HC. This is in line with PG and SUD research. Reduced LA has been found before in slot machine gamblers^61^. In our PG cohort 17 of 19 subjects indicated slot machines as their primary gamble. So the behavioral part of our study may be seen as a replication of that study. Another study observed no mean difference in LA between PG and HC, but instead some PG subjects with very high and some with very low LA^14^. The PG group in that study had already undergone PG treatment while our PG subjects were active gamblers with little to no treatment. This may be the reason why in our sample LA in PG is significantly lower than in the HC sample. Also the study by Gelskov *et al*.^26^ have found only a trend in LA difference between PG and HC subjects. Yet, also their PG subjects had undergone PG treatment. Also the study by Giorgetta *et al*.^13^ has found an increase in LA with amount treatment received, while Brevers *et al*.^12^ have observed significantly reduced LA in active gamblers who had not received treatment. These results indicate that PG treatment may lead to a normalization of LA in PG subjects.

Both PG and AD patients showed reduced LA due to reduced behavioral loss sensitivity while behavioral gain sensitivity was not different compared to HC subjects in both groups. To our knowledge, our study is the first to report reduced LA in AD patients, comparable to reduced LA in PG subjects. Further, our study seems to be the first reporting on the basis for reduced LA, namely reduced behavioral loss sensitivity, concurrently in both a SUD sample and a PG sample. Previous LA studies in PG have made no statements on differences in behavioral gain and loss sensitivity to try to explain reduced LA in PG subjects.

We further hypothesized that LA would be correlated with symptom severity. We saw within PG subjects that the higher their LA, the lower they scored on the GBQ, i.e. gamblers’ beliefs. The correlation with the GBQ suggests that low LA in PG subjects is related to higher cognitive distortions, such as illusions of control (“I can control the outcome of the gamble”) and beliefs of persistence (“If I lose I should keep gambling to not miss out on any wins.”). However, within AD patients, we did not find a correlation with any AD severity score. This may indicate that the LA task is better suited for severity assessment in PG than in AD subjects. One reason for this may be that the LA task itself is a gambling task capturing core features of the addictive behavior and its consequences for PG subjects (e.g. relative immediacy of losses in the financial domain) but less so for AD subjects.

With respect to neural loss sensitivity we expected stronger DLPFC deactivation in response to rising losses in HC subjects compared to both clinical groups. We indeed observed this in AD subjects. However, the significant HC < AD contrast was also due to the fact that AD subjects showed a widespread increase in lateral prefrontal activity with rising losses. Hence it seemed that AD subjects with rising losses actually recruited increasing cognitive resources in DLPFC, while HC subjects stayed put or even decreased activity. Also reaction times pointed into that direction: AD subjects became slower with rising losses while HC subjects did not change their reaction times. PG subjects did not significantly differ in their DLPFC activity in the face of rising losses. In that sense AD and PG seem to differ. However, direct comparison of PG and AD subjects failed to reach significance. Future studies should readdress this direct comparison with larger sample sizes.

AD subjects also showed larger activity increase with rising losses in VLPFC. This effect may stem from the task structure, which had a speedy reaction component and, since responses were always mapped to the same buttons, also an inhibition component. With DLPFC and VLPFC activating despite high losses, AD subjects thus may have employed more working-memory^62,63^ and cognitive control^40,41,64,65^ compared to HC subjects when high losses were at stake. PG subjects seemed not to differ from HC subjects, but also not clearly from AD subjects. However, note that like AD subjects also PG subjects increased response speed with rising gains and reduced it with rising losses. And the study by Gelskov *et al*. (2016) has shown higher DLPFC activity during unfavorable gambles in PG subjects in a similar decision-making task^26^. This points to increased employment of cognitive resources despite high losses similar to AD subjects. Alterations in working memory have been linked before to alterations in decision-making in SUD cohorts^66^. Further, higher DLPFC and parietal activity has been associated with higher risk taking in binge drinkers vs. HC^67^, and dysfunctions of the DLPFC may lead to cognitive inertia^68^.

Our study was guided by network models also offering neural connectivity explanations for inter-individual differences in LA^27,30^. Using these models as hypothesis generators we explored functional connectivity differences between the groups, because they may well be an additional basis to explain group differences in LA. Only PG subjects showed reliable altered functional connectivity. They showed a stronger gain-related functional connectivity from amygdala to posterior OFC compared to HC. According to the ANDREA model^27^, this may mean that amygdala enhances the representation of gain values in the OFC. This may lead to decreased LA because losses are becoming less salient with rising gains. We further saw that the functional loss-related connectivity between amygdala and VMPFC^30^ was stronger in HC subjects than in PG subjects. This perhaps points to decreased production of loss-related salience signals in PG subjects, possibly disturbing proper cost-benefit evaluation. Similarly, PG subjects’ functional connectivity from OFC to DRN was weaker compared to HC subjects. Since DRN is hypothesized to code for negative time difference prediction errors by receiving value signals from OFC^27,69,70^, this may mean that PG subjects forward loss signals less efficiently compared to HC subjects. Our results support the notion that pathological gambling might be associated with changes in task-relevant communication between brain areas of the reward system^71,72^.

### Limitations

Our study must be interpreted with caution. Small sample sizes and a large NOI limited statistical power. Our exploratory analyses have to be backed by greater sample sizes and completely controlled for multiple testing in the future. However, our study is innovative because we have directly compared an SUD and a behavioral addictive disorder and we have used an extensive set of tools to investigate the neural correlates of reduced LA in PG and AD. Disentangling the psychological from the neurotoxic factors of addiction is one of the great challenges of current neurobehavioral research^73^. Further comparative and transdiagnostical studies like ours are needed to find neurobehavioral markers for etiology research, for better diagnosis and better measurement of treatment success^3,74–77^. Good matching is key to such studies. Our matching was imperfect with respect to age, however we checked all our results for stability of results by statistical adjustment procedures. Further, PG and AD subjects with no comorbidities may hamper generalizability. However, we were interested in isolating basic neurobiological mechanisms. Hence, isolating the disorder in question and not allowing additional diagnoses introduce more variance was apt here. Debt is an integral part of PG disorder^78^ and it co-varied with LA (see Supplementary Information). Future studies could focus on this issue and associate financial decisions, LA, debt and gambling symptoms^79,80^. We have further only considered male PG and male AD subjects. A bias for male subjects is common in the gambling literature^8^. Female PG subjects are less prevalent^81^. Further, sex differences in LA are known^82^. Here, we wanted to limit variance and thus focused on only one gender. Future studies should address sex differences in impaired decision-making in PG subjects. Moreover, the current study is not designed to disentangle differences in loss aversion completely from differences in risk aversion. Future studies should address this, e.g. by orthogonalizing variance and expected value, by varying probability of gains and losses and by introducing gain and loss-only trials.

## Conclusions

We have observed reduced LA in both PG and AD subjects. In both groups, this reduction was due to reduced behavioral loss sensitivity. AD subjects showed altered loss-related DLPFC and VLPFC reactivity. It was unclear whether PG subjects differed in this regard from AD subjects or HC subjects. In exploratory analyses PG subjects showed enhanced gain-related amygdala-OFC connectivity, reduced loss-related amygdala-VMPFC and OFC-DRN connectivity. The neural differences to HC subjects might reflect disturbed cost-benefit calculations when assessing gambles in both PG and AD subjects. However, the neural processes leading to this reduction in LA in both PG and AD might be different. LA correlated with symptom severity only within PG subjects. Accordingly, the increase of LA has been related to PG therapy^12,13^. The LA task and its neural correlates may thus prove valuable for diagnosis and treatment of PG. The LA task may be remodeled into a training tool to augment behavioral therapy^83^. Such a computerized application could teach to properly anticipate losses and to disengage from gambling if losses hit a certain threshold regardless of possible gains. This may be paired with learning to feel rewarded by successful loss avoidance^84^.

## Electronic supplementary material

Supplementary Information

Network of Interest mask
