# Supplementary material for: Reduced loss aversion in pathological gambling and alcohol dependence is associated with differential alterations in amygdala and prefrontal functioning
Source: Sci Rep. 2017 Nov 24;7:16306. doi: 10.1038/s41598-017-16433-y (PMC5701119; doi:10.1038/s41598-017-16433-y)
Supplement: Supplementary file 1 — Supplementary Information [file 41598_2017_16433_MOESM1_ESM.pdf]

Supplementary Materials

Title of article:

Reduced loss aversion in pathological gambling and alcohol dependence is associated with differential alterations in amygdala and prefrontal functioning.

Authors:

Alexander Genauck[1)2)], Saskia Quester[1)3)], Torsten Wüstenberg[1)], Chantal Mörsen[1)], Andreas Heinz[1)], Nina Romanczuk-Seiferth[1)]

Affiliations:

1) Department of Psychiatry and Psychotherapy, Charité Universitätsmedizin Berlin
2) Bernstein Center for Computational Neuroscience Berlin
3) Berlin School of Mind and Brain, Humboldt-Universität zu Berlin

Corresponding author:

Alexander Genauck, Department of Psychiatry and Psychotherapy, Charité - Universitätsmedizin Berlin, Charitéplatz 1, 10117 Berlin, Germany, +49 30 450 517-307, alexander.genauck@charite.de

CONTENTS

1 Supplementary methods 3

1.1 Administered questionnaires 3

1.2 Reaction times 4

1.3 The behavioral model 5

1.4 MRI data acquisition and preprocessing 8

1.5 The fMRI single-subject model 10

1.6 The network of interest (NOI) 11

1.7 The rBPM analysis 14

1.8 Functional connectivity 15

2 Supplementary results 17

2.1 Reaction times 17

2.2 Debt 19

2.3 Functional connectivity 20

2.4 Voxel-based morphometry 21

2.5 References 23

1 Supplementary methods

1.1 Administered questionnaires

PG subjects were diagnosed using the German short questionnaire for gambling behavior questionnaire (Kurzfragebogen Spielsucht, KFG) (cutoff $\geq$ 16) (Petry and Baulig, 1996), internal consistency, i.e. Cronbach's Alpha = 0.79, retest reliability 2 weeks = 0.80 (Petry, 1996). According to the KFG 4 subjects displayed mild, 14 subjects medium and 1 subject severe PG. Otherwise, any known history of a neurological disorder or a current psychological disorder (except substance abuse and tobacco dependence) as assessed by the screening of the Structured Clinical Interview for DSM-IV Axis I Disorders (SCID-I) (First *et al*, 2002) lead to exclusion from the study. For matching purposes subjects completed the Wechsler Intelligence Test for Adults (WAIS) matrices test (Wechsler, 1997) and they were asked to indicate age, smoking status, amount of personal debt and monthly personal income. Furthermore, they were asked to indicate their level of education and handedness (Oldfield, 1971). For further characterization of the three groups subjects also completed Beck's Depression Inventory (BDI-II) (Beck *et al*, 1996) and the Barratt Impulsiveness Scale Version 10 (BIS-10) (Patton *et al*, 1995).

1.2 Reaction times

Reaction times were submitted into a linear mixed effects model with random effects (Bates *et al*, 2015b), where centralized gain, centralized loss, absolute Euclidean distance were fixed effects and also allowed to vary randomly per subject, using the lmer function in lme4 in R. In a second model fixed effects of gain and loss were modulated by group. Both models were compared using the anova function in R performing a Chi-Square-Difference test. Post-hoc t-tests were performed using Satterthwate's approximations implemented in lmerTest (Kuznetsova *et al*, 2016).

1.3 The behavioral model

Several models (within subject) were considered to model the behavioral data. The model by (Tom *et al*, 2007) but with Euclidean distance (**lae**, $value = \beta 0 + \beta_{gain} * gain + \beta_{loss} * loss + \beta_{ed} * ed$), the original model by (Tom *et al*, 2007) used in the current study (**la**, $value = \beta 0 + \beta_{gain} * gain + \beta_{loss} * loss$), the ratio model (Gelskov *et al*, 2016) (**lar**, $value = \beta 0 + \beta_{ratio} * ratio$) and the De Martino/Charpentier model (Charpentier *et al*, 2015; De Martino *et al*, 2010) (**lac**, $value = 1 * gain + \lambda * loss$). Value was subjected to a two-options softmax function $P(accept = 1) = (1 + \exp(-\mu * value))^{-1}$ (Charpentier *et al*, 2015; Sutton, 1998) with $\mu = 1$ (logistic function) or with μ as a free parameter (choice consistency, i.e. for model **lac**). Note that **lac**'s value function can be rewritten as $value = \mu * gain + \mu * \lambda * loss$, which then is submitted to the logistic function without any free parameter. **Lac** is hence a logistic regression like **la** but without an intercept $\beta 0$, with $\beta_{gain} = \mu$ and $\beta_{loss} = \mu * \lambda$ (hence $\lambda = \beta_{loss} / \beta_{gain}$). Inversely, in **la**, **lae** β_{gain} may be seen as μ because one can write for **la** (and accordingly for **lae**) $value = (\beta 0 + 1 * gain + \lambda * loss) * \mu$, from which follows $\beta_{gain} = \mu$ and $\beta_{loss} = \lambda * \mu$ and hence again $\lambda = \beta_{loss} / \beta_{gain}$. To perform model comparison we estimated each model using the glmer function in lme4 (Bates *et al*, 2015b) in each group separately or with all groups together using group as a between subject fixed effect, respectively. From the glmer models we could simply note down the Aikaike Information Criterion (AIC) values and computed mean AIC values, so that all reported AIC values are always "mean AIC per subject" values. Only this way AIC values can be compared between groups, because the groups have different sizes (**Table S1**).

The **la** model had the lowest mean group AIC value (i.e. best model), also reflected in the likelihood ratio tests comparing all models to **lae** (**Table S1**). We thus chose for the analyses in the main text the **la** model. We used mixed effects modeling because it yields more robust single-subject parameter estimates and also mixed effects modeling is designed to estimate group fixed effects (Bates *et al*, 2015a).

We computed $\lambda's$ per model and correlated them. The lambdas of **la** correlated well with λ's of all other considered models (**Table S2**). We also computed mean λ values per group and model and performed group comparisons. For this we extracted the fixed effects and random effects and added them and computed one λ values per subject and model. Note that this is a different but very fast method to estimate the fixed effect of loss aversion (λ) and get standard errors of the parameters. This method was only used here, not in our main analysis, where we bootstrapped parametrically the p-values for group comparisons of fixed effects of λ. Note that all models yielded the same expected group differences (**Table S3**).

Table S1: AIC values of different LA models split by group

Model	df	HC	PG	AD	mean of group AICs	complete model AIC	Likelihood ratio test against lae
**lae**	22	91.7	103.1	127.9	107.5	106.81	-
**la**	15	91.5	102.6	127.8	107.3	106.78	ΔLL = -6.2, Δdf = -7, 0.084
**lar**	9	99.4	107.2	134.1	113.6	112.63	ΔLL = 161.4, Δdf = -13, p<0.001
**lac**	12	140.0	132.1	152.1	141.4	135.09	ΔLL = 1421, Δdf = -20, p<0.001

ΔLL is difference in log-likelihood, negative values mean it is a better fitting model than lae, positive values mean it is a worse fitting model; df is degrees of freedom of complete model; Δdf is the difference in degrees of freedom, i.e. difference in estimated parameters;

Table S2: Spearman correlation coefficients of λ estimates of different models across all groups.

λ	lae	la	lar	lac
**lae**	1	0.99	0.6	0.95
**la**		1	0.53	0.98
**lar**			1	0.43
**lac**				1

0 means n.s. Spearman correlation

Table S3: Mean λ values per group and model.

	HC	PG	AD	p(HC > PG)	p(HC > AD)	p(PG > AD)
**lae**	2.33	1.09	1.16	**0.020**	**0.030**	0.789
**la**	2.29	1.15	1.21	**0.028**	**0.040**	0.804
**lar**	2.04	1.64	1.62	**0.018**	**0.012**	0.905
**lac**	2.19	1.19	1.26	**0.032**	**0.049**	0.761

p’s are p-values of two-sample t-tests

1.4 MRI data acquisition and preprocessing

Scanning was performed with a 3-Tesla clinical whole-body magnetic resonance tomograph (MR Magnetom Tim Trio, Siemens, Erlangen, Germany) equipped with a standard 12-channel phased-array head coil at Charité – Universitätsmedizin Berlin. In the T2*-sensitive Gradient-Echo Echo-Planar Imaging (GE-EPI) sequence used during the loss aversion (LA) task, 39 slices covering the whole brain were acquired in an interleaved order and ascending acquisition direction (TR=2.5s, 3mm thickness, 0.5mm inter-slice gap, TE: 35ms, flip angle: 80°, in-plane resolution: 64 x 64 pixels, voxel size: 3.5mm x 3.5mm x 3.0mm). Before the GE-EPI sequence, a T1-weighted 3D structural image for anatomical referencing (Magnetization Prepared Rapid Gradient Echo, MPRAGE, voxel size: 1mm x 1mm x 1mm) and a B0 fieldmap for image distortion correction were recorded. Imaging data were processed with Statistical Parametric Mapping (SPM12, http://www.fil.ion.ucl.ac.uk/spm/, Wellcome Department of Imaging Neuroscience, London, UK) running on MATLAB (version: R2014a, Mathworks, Sherborn, MA, USA). The GE-EPI images of every subject were corrected for differences in slice acquisition time. GE-EPI images were registered to the mean GE-EPI image. Fieldmaps were used to unwarp non-linear image distortions caused by B0 inhomogenities (Andersson *et al*, 2001). The T1 image was co-registered to the unwarped mean GE-EPI image using affine spatial transformation. The T1 image was then segmented into tissue classes and transformed into the Montreal Neurological Institute-standard space (MNI). This process yielded linear and non-linear parameters for the transformation between individual and standard space, which were applied to all unwarped EPI images. EPI images were resampled to a voxel size of 3.5mm x 3.5mm x 3.5mm. Finally, these images were spatially smoothed with an isotropic Gaussian

kernel (full-width-at-half maximum 8mm). Additionally, we used the VBM8 toolbox (Kurth *et al*, 2010) to segment T1 images into tissue classes. Gray matter tissue probability maps (TPMs) were than warped into standard space, spatially smoothed and down sampled to a voxel size of 3.5mm x 3.5mm x 3.5mm to match the resolution of our functional images. These gray matter TPMs then represent local gray matter volume or local gray matter density (GMD) (Good *et al*, 2002), irrespective of overall brain size, and lend themselves for BPM analysis.

1.5 The fMRI single-subject model

Additionally, the head motion parameters obtained during motion correction were entered into the model to account for signal fluctuations caused by the interaction of movement and susceptibility (Morgan, Dawant, Li, & Pickens, 2007). After high pass filtering (cut off frequency = 1/128 Hz) and the elimination of high frequency noise by autoregressive (AR(1)) modeling, the General Linear Model (GLM) was fit to the preprocessed EPIs using a restricted maximum likelihood algorithm. Only gray matter voxels according to the SPM12 gray matter template ($p > 0.2$) were considered.

1.6 The network of interest (NOI)

Our NOI consisted of cortical and limbic areas derived from relevant LA and related studies (Aron *et al*, 2014; Basten *et al*, 2010; Canessa *et al*, 2013; De Martino *et al*, 2010; Gelskov *et al*, 2016; Levy and Wagner, 2011; Litt *et al*, 2008; Sokol-Hessner *et al*, 2013; Tom *et al*, 2007; Venkatraman *et al*, 2011). Specifically, the NOI included caudate, putamen, accumbens region, VLPFC, medial frontal cortex for ventral medial prefrontal cortex (VMPFC), orbital gyrus, amygdala, anterior cingulate, insula, DLPFC, ventral tegmental area/midbrain (VTA), dorsal raphe nucleus (DRN). The NOI was created using labels according to the SPM12's Neuromorphometrics Inc. atlas (see **Table S5**).

Table S4: Overview of SPM12 ROIs used for NOI.

SPM12's atlas label	Also referred to as
Right Accumbens Area	ventral striatum (VS)
Left Accumbens Area	ventral striatum (VS)
Right Putamen	
Left Putamen	
Right Caudate	
Left Caudate	
Right ACgG anterior cingulate gyrus	
Left ACgG anterior cingulate gyrus	
Right Amygdala	
Left Amygdala	
Right AOrG anterior orbital gyrus	
Left AOrG anterior orbital gyrus	
Right LOrG lateral orbital gyrus	
Left LOrG lateral orbital gyrus	
Right MOrG medial orbital gyrus	
Left MOrG medial orbital gyrus	
Right POrG posterior orbital gyrus	
Left POrG posterior orbital gyrus	
Right MFC medial frontal cortex	ventral medial prefrontal cortex (VMPFC)
Left MFC medial frontal cortex	ventral medial prefrontal cortex (VMPFC)
Right AIns anterior insula	
Left AIns anterior insula	
Right PIns posterior insula	
Left PIns posterior insula	

For VTA we used a probabilistic ROI of the midbrain (Murty *et al*, 2014). These authors constructed a midbrain mask based on hand-drawn VTA-substantia-nigra-midbrain masks of 50 healthy subjects. For DRN we used an 8mm radius sphere around the MNI coordinate [-2, -32, -16] (Pedroni *et al*, 2011). Note that both areas are quite large with respect to the actual size of the mentioned nuclei to account for inter-individual differences. These masks for VTA and DRN were chosen because these areas are not part of the SPM12 atlas, nor the AAL atlas. For DLPFC we used the WFU pick atlas to select Brodman areas (BA) 8,9,10 and 46 (dilated in 2D, i.e. in-plane, by 1 voxel) (Collins, 2001; Draganski *et al*, 2008; Maldjian *et al*, 2003) within the middle frontal gyrus according to the AAL atlas (Tzourio-Mazoyer *et al*, 2002). For VLPFC we used BA 44, 45, 47 (dilated in 2D, i.e. in-plane, by 1 voxel) within the inferior frontal gyrus (Badre and Wagner, 2007; Danker *et al*, 2008; Gold *et al*, 2006) (**Figure S1**). The complete NOI can be found as .nii file in the Supplementary Online Material.

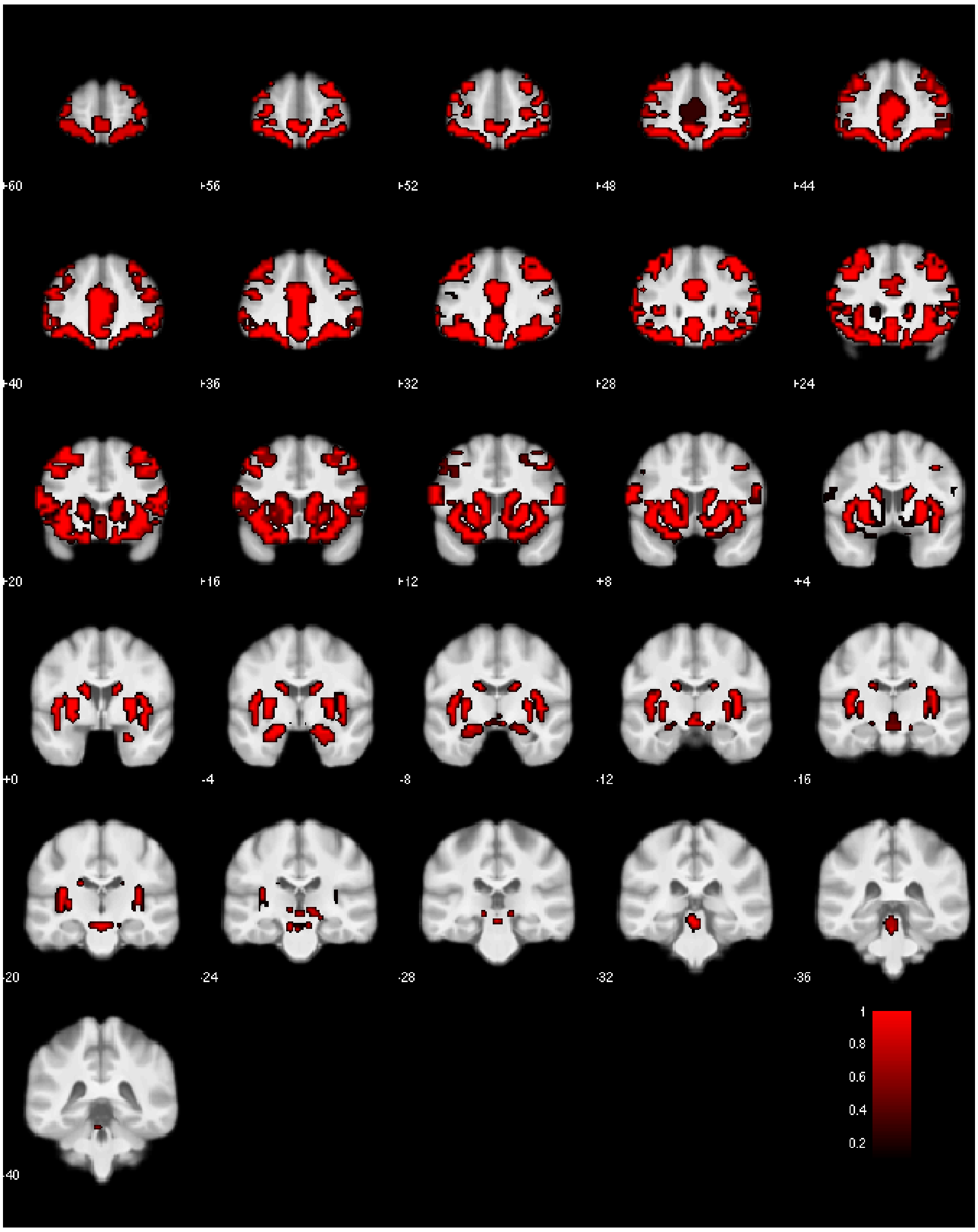

Figure S1: Network of interest (NOI). Mask (red) superimposed on mean of normalized structural T1-images of all subjects**.** Slices are shown from y = +60 to y = -40 in steps of -4 (top to bottom). Regions were taken from SPM12 Neuromorphometrics atlas, as well as AAL and BA atlas within the wfu pick atlas (DLPFC and VLPFC), as well as external sources (midbrain, DRN). Regions were selected based on literature sources reporting on the neural correlates of inter-individual differences in loss aversion tasks. This NOI mask was used for small volume correction in group comparisons of neural gain and loss sensitivity as well as for other exploratory analyses.

1.7 The rBPM analysis

Robust biological parametric mapping (rBPM in toolbox BPMe) was used running on SPM5 (Casanova *et al*, 2007; Yang *et al*, 2011) and results were evaluated in SPM8. Note that BPMe is only available for SPM5 but results may be evaluated in SPM8 but not in SPM12.

1.8 Functional connectivity

We fit new single subject models. Specifically, for every seed region we expanded the standard single subject model by interaction terms multiplying the time series of the respective seed region and each parametric modulator (McLaren *et al*, 2012). All the other terms in the single subject model, including motion parameters as covariates of no interest, stayed the same. We then submitted the contrast images pertaining to the interaction terms for gain and loss to second-level T-tests comparing PG and AD to HC, respectively.

In the ANDREA model (**Fig. S2**), when LA exists, the amygdala sends a salience signal to OFC which is stronger for losses than for gains. This enhances the represented loss value over the represented gain value in OFC. Lack of LA may thus emerge from a more efficacious transmission of the amygdala salience signal for gains. We thus expected a functional connectivity which grows more strongly for increasing gains in both PG and AD subjects compared to HC subjects.

According to (Basten *et al*, 2010) (**Fig. S2**), the VMPFC is said to be a comparator region integrating cost signals from amygdala and gain signals from VS. We hence computed a gPPI analysis on single subject level with amygdala as seed region and used the VMPFC ROI for small volume correction and expected HC to show stronger functional connectivity from amygdala to VMPFC with respect to growing losses than both PG and AD subjects.

Found group differences in functional connectivity were checked for stability against adjusting for age using ancova analysis in SPM. Only results are reported which survived adjustment for age.

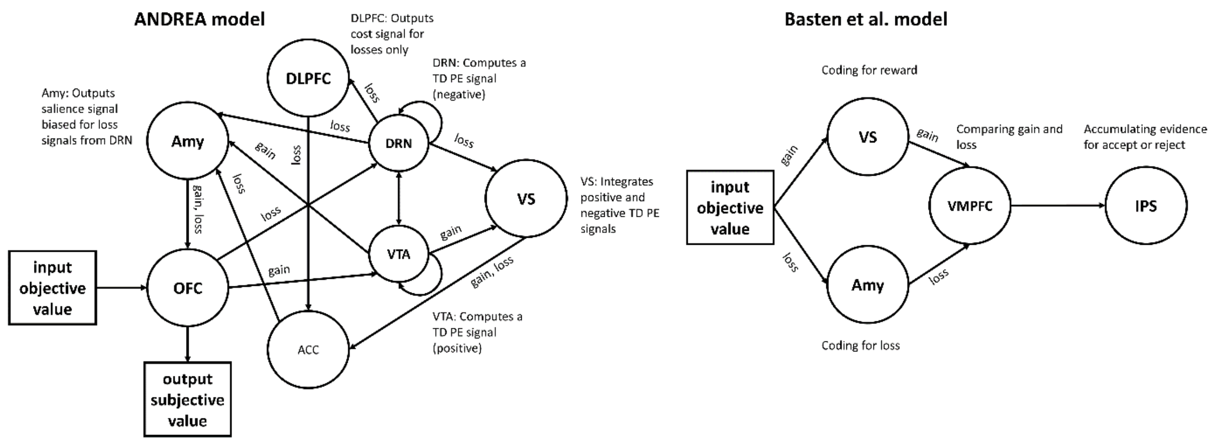

Figure S2: Network models for gPPI analyses. The ANDREA model (left, adapted from (Litt *et al*, 2008)) and the model by Basten et al. (right, adapted from (Basten *et al*, 2010). The network models were used as hypotheses generators regarding differences in functional connectivity between PG, AD and HC subjects. The arrows mean functional connections. Next to the arrows it is stated whether the connection processes gain or loss signals.

2 Supplementary results

2.1 Reaction times

Inclusion of group into the behavioral model was significant, $\Delta df = 6$, $p(\Delta Chi^2) = 0.023$, $\Delta AIC = 2$. The HC group showed a mean reaction time (rt) of 1.27s, the AD group of 1.54s and the PG group of 1.39s. HC's rt was shorter than that of AD subjects (HC < AD, $p = 0.030$). AD patients showed a stronger increase in rt with growing losses than HC subjects ($\beta = 0.019$, $p = 0.019$), also PG subjects showed this ($\beta = 0.018$, $p = 0.018$). With increasing gains, PG subjects showed a stronger decrease in rt compared to HC ($\beta = -0.011$, $p = 0.033$) (**Figure S3**). Adjusting for age by allowing age to impact the fixed intercept and the rt within each group, yielded the same results, except the overall mean difference in rt of HC vs. AD and HC vs. PG was rendered insignificant.

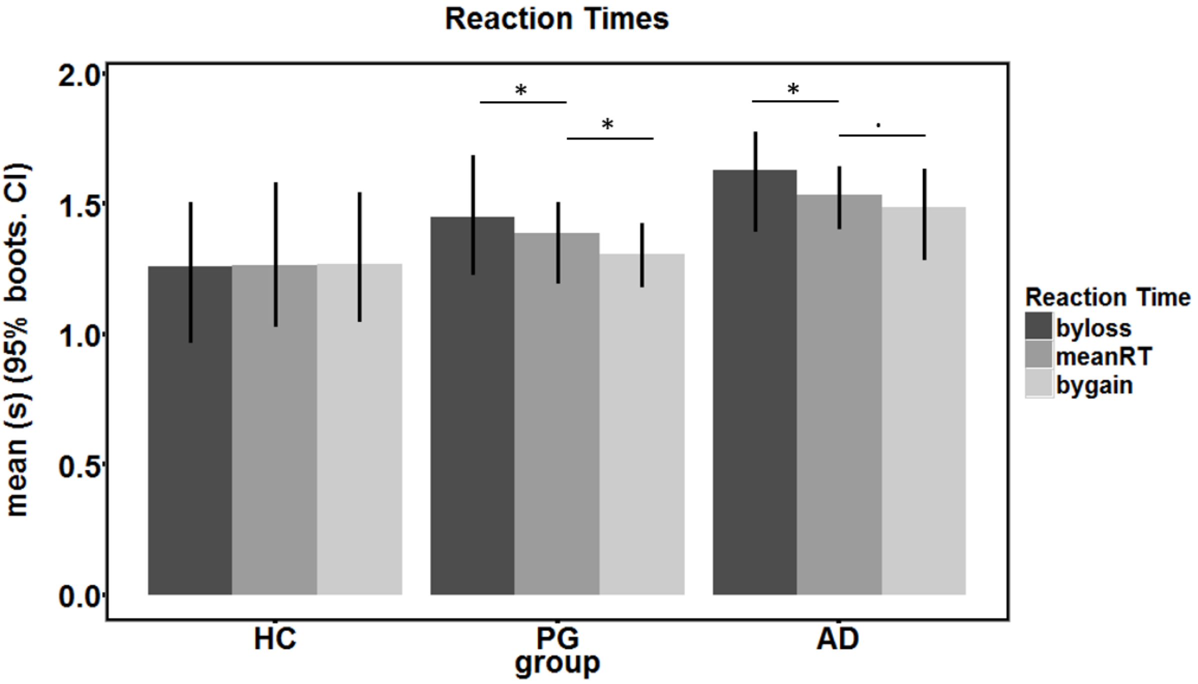

Figure S3: Reaction times. Depicted are mean reaction times (time until decision is made) per group and condition in seconds with bootstrapped 95% confidence intervals. MeanRT is the mean reaction time at presentation of mean gain and mean loss in the proposed gamble. Bygain shows how this meanRT changes when gain increases by 5 euros. Byloss shows how reaction time changes when losses increase by 5 euros. Note that PG and AD subjects change their reaction times as a function of gain and loss but not HC subjects.

2.2 Debt

We have checked the relationship of debt (yes/no) (28 yes, 19 no, 4 NA) and loss aversion. The median LA for no debt was 1.64 and for debt 0.97. This difference was significant (Kruskall-Wallis test, $p = 0.02$). We fit our original model (group explaining behavioral gain and loss sensitivity) and the alternative model (debt (yes/no) explaining behavioral gain and loss sensitivity), while excluding in both cases the 4 subjects which did not provide information on their debt. Model comparison showed that the group model was still slightly better than the debt model: $\Delta df = 4$, $Chi^2 = 11.4$, $p = 0.022$, $\Delta AIC = 3.5$ (AIC of group model better than that of debt model). We could not usefully correlate the amount of debt with behavioral LA because we had too many missings (15 NA) in the variable "amount of debt". This is because 15 subjects declined to answer this question.

2.3 Functional connectivity

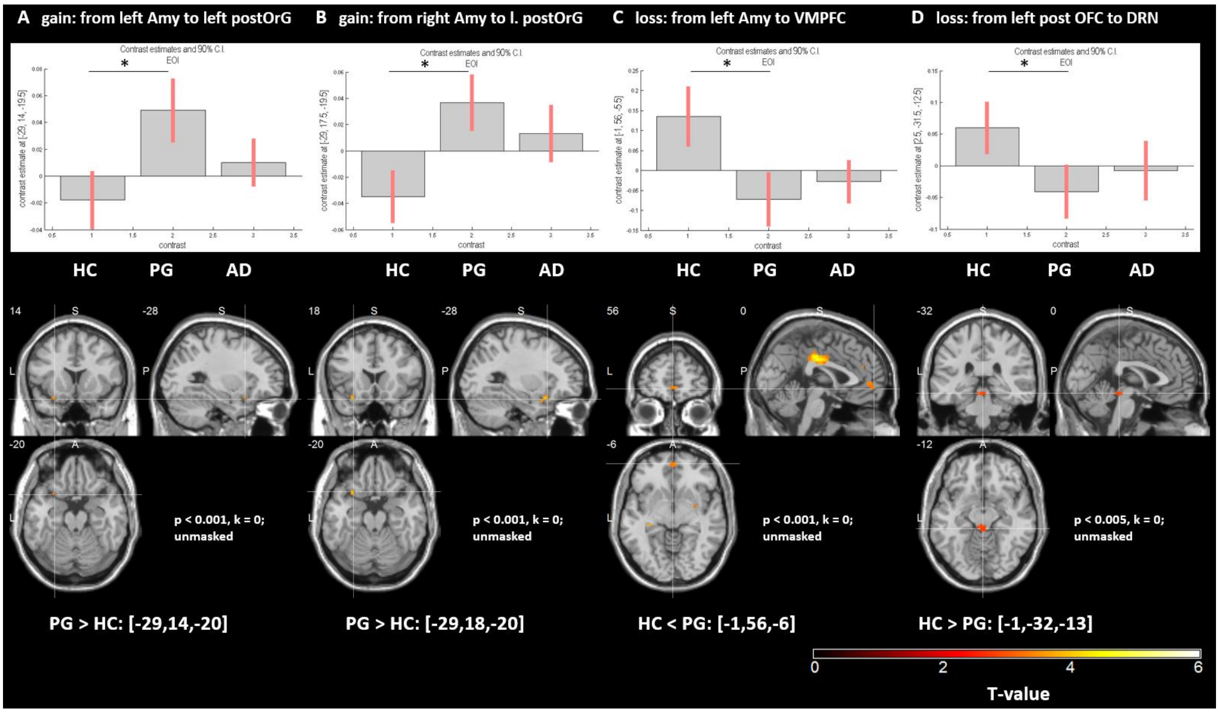

Figure S4: Functional connectivity group differences. A,B: PG subjects show a stronger functional connectivity from Amygdala to posterior OFC with regards to growing gains. It seems they transmit the amygdala signal with respect to gains more and more efficaciously to OFC, when gains increase, while HC subjects do so less or even do the reverse. **C:** With growing losses HC subjects show stronger connectivity increase from left amygdala to VMPFC than PG subjects. It seems they transmit the amygdala signal with respect to losses more and more efficaciously to OFC, when losses increase, while HC subjects do so less. **D:** The same is true for the functional connectivity from left posterior OFC to DRN.

2.4 Voxel-based morphometry

We checked functional results for stability against adjusting for local gray matter density (GMD) and age. Here we contrast GMD maps (adjusted for age) to display the GMD differences between clinical groups (PG, AD) and HC. We look at contrasts **HC > PG, HC < PG**, **HC > AD, HC < AD, PG > AD, AD < PG,** with the latter two masked by the F-conjunction **HC <> PG, HC<>AD, PG<>AD** (**Figure S5**). We explore at $p < 0.001$, uncorrected, $k = 10$, apply small volume correction using our NOI at $p_{SVC} = 0.05$, and apply whole brain FWE correction at $p_{FWE} = 0.05$.

HC > PG yielded no suprathreshold voxels. SVC and whole brain correction yielded no results. HC < PG yielded three major clusters: at left parietal operculum / supramarginal gyrus, [-54, -42, 23], at DLPFC, i.e. left middle frontal gyrus [-26, 49, 16] and at ACC [3, 32, 19]. SVC NOI and whole brain FWE correction yielded no significant peak voxels. HC < AD yielded no suprathreshold voxels, SVC NOI and whole brain FWE correction yielded no significant voxels. HC > AD yielded major clusters at right precentral gyrus [27, 25, 58], right medial orbital gyrus [13, 32, -30] and right supramarginal gyrus [48, -28, 44] and right cerebellum [38, -49, -34]. SVC NOI and whole brain FWE correction yielded no sig. voxels. PG > AD (masked by F-conjunction) yielded a cluster in right middle cingulate gyrus, [6,15,37], and right precentral gyrus [40,-19,37]. SVC NOI and whole brain FWE correction yielded no sig. voxels. PG < AD (masked by F- conjunction) yielded no significant voxels, neither when applying whole brain and SVC NOI correction, nor on exploratory level.

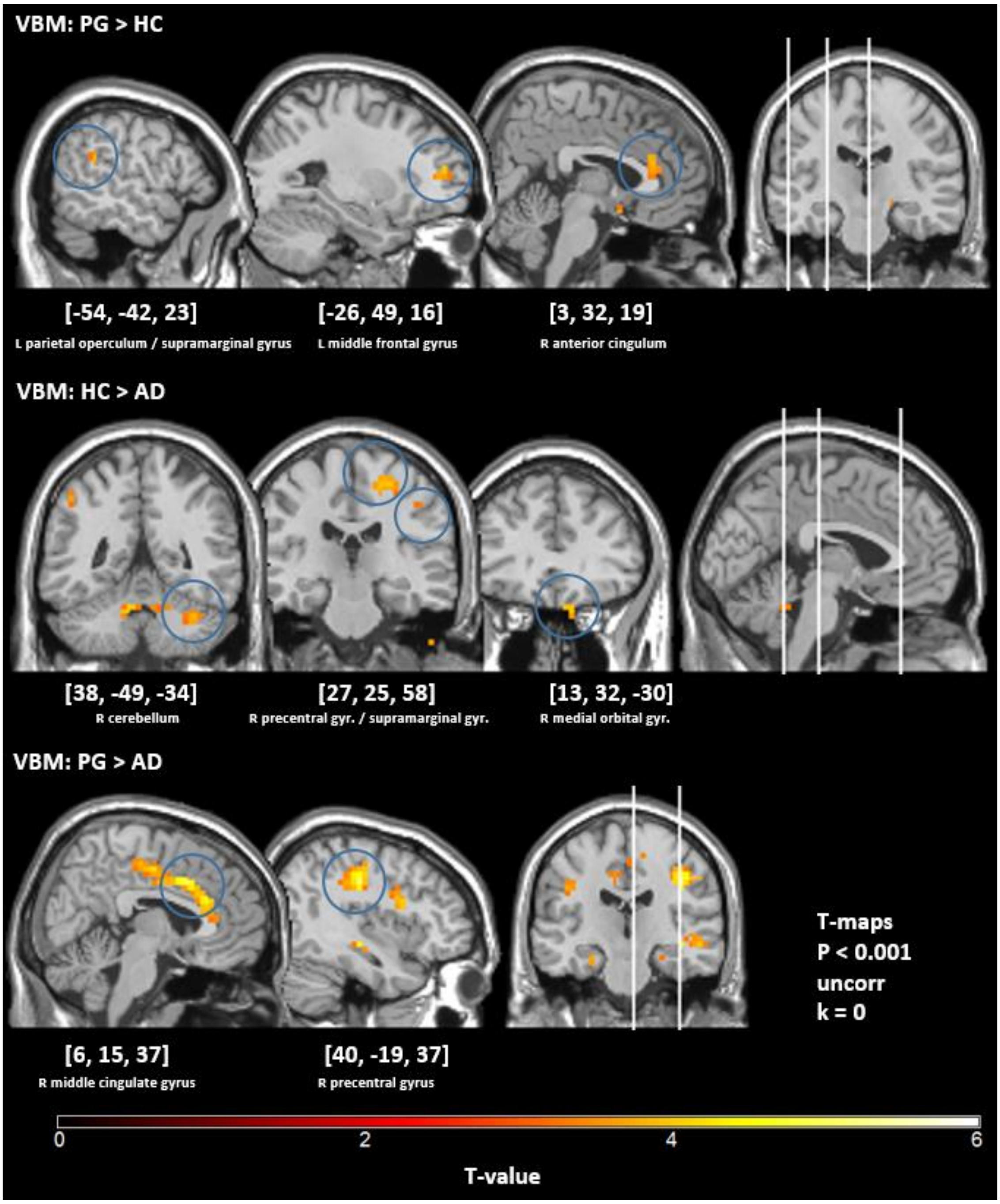

Figure S5: Voxel-based morphometry (VBM) analysis. Results of one-way ANOVA adjusted for age. First line of images shows VBM contrast of PG > HC at p < 0.001, k = 0. Second line of images shows VBM contrast of HC > AD at p < 0.001, k = 0. Third line of images shows VBM contrast of PG > AD at p < 0.001, k = 0.

2.5 References

Andersson JLR, Hutton C, Ashburner J, Turner R, Friston K (2001). Modeling Geometric Deformations in EPI Time Series. *NeuroImage* **13**: 903–919.

Aron AR, Robbins TW, Poldrack RA (2014). Inhibition and the right inferior frontal cortex: one decade on. *Trends Cogn Sci* **18**: 177–185.

Badre D, Wagner AD (2007). Left ventrolateral prefrontal cortex and the cognitive control of memory. *Neuropsychologia* **45**: 2883–2901.

Basten U, Biele G, Heekeren HR, Fiebach CJ (2010). How the brain integrates costs and benefits during decision making. *Proc Natl Acad Sci* **107**: 21767–21772.

Bates D, Mächler M, Bolker B, Walker S (2015a). Fitting Linear Mixed-Effects Models Using lme4. *J Stat Softw* **67**: 1–48.

Bates D, Maechler M, Bolker B, Walker S (2015b). lme4: Linear mixed-effects models using Eigen and S4. *R Package Version 11-8* at <http://keziamanlove.com/wp-content/uploads/2015/04/StatsInRTutorial.pdf>.

Beck AT, Steer RA, Brown Gk (1996). BDI-II, Beck depression inventory: manual: Psychological Corp. *San Antonio TX* .

Canessa N, Crespi C, Motterlini M, Baud-Bovy G, Chierchia G, Pantaleo G, *et al* (2013). The Functional and Structural Neural Basis of Individual Differences in Loss Aversion. *J Neurosci* **33**: 14307–14317.

Casanova R, Srikanth R, Baer A, Laurienti PJ, Burdette JH, Hayasaka S, *et al* (2007). Biological parametric mapping: a statistical toolbox for multimodality brain image analysis. *Neuroimage* **34**: 137–143.

Charpentier CJ, Martino BD, Sim AL, Sharot T, Roiser JP (2015). Emotion-induced loss aversion and striatal-amygdala coupling in low-anxious individuals. *Soc Cogn Affect Neurosci* nsv139doi:10.1093/scan/nsv139.

Collins ML (MIT Press: 2001). *Handbook of Developmental Cognitive Neuroscience*. .

Danker JF, Gunn P, Anderson JR (2008). A rational account of memory predicts left prefrontal activation during controlled retrieval. *Cereb Cortex* **18**: 2674–2685.

De Martino B, Camerer CF, Adolphs R (2010). Amygdala damage eliminates monetary loss aversion. *Proc Natl Acad Sci U S A* **107**: 3788–3792.

Draganski B, Kherif F, Klöppel S, Cook PA, Alexander DC, Parker GJM, *et al* (2008). Evidence for Segregated and Integrative Connectivity Patterns in the Human Basal Ganglia. *J Neurosci* **28**: 7143–7152.

First MB, Spitzer RL, Gibbon M, Williams JB (2002). Structured clinical interview for DSM-IV-TR axis I disorders, research version, patient edition. *N Y Biom Res N Y State Psychiatr Inst* .

Gelskov SV, Madsen KH, Ramsøy TZ, Siebner HR (2016). Aberrant neural signatures of decision-making: Pathological gamblers display cortico-striatal hypersensitivity to extreme gambles. *c* **128**: 342–352.

Gold BT, Balota DA, Jones SJ, Powell DK, Smith CD, Andersen AH (2006). Dissociation of automatic and strategic lexical-semantics: functional magnetic resonance imaging evidence for differing roles of multiple frontotemporal regions. *J Neurosci* **26**: 6523–6532.

Good CD, Johnsrude IS, Ashburner J, Henson RN, Fristen KJ, Frackowiak RS (2002). A voxel-based morphometric study of ageing in 465 normal adult human brains. *Biomed Imaging 2002 5th IEEE EMBS Int Summer Sch On* 16–ppat <http://ieeexplore.ieee.org/abstract/document/1233974/>.

Kurth F, Luders E, Gaser C (2010). VBM8 toolbox manual. *Jena Univ Jena* .

Kuznetsova A, Brockhoff PB, Christensen RHB (2016). *lmerTest: Tests in Linear Mixed Effects Models*. at <http://CRAN.R-project.org/package=lmerTest>.

Levy BJ, Wagner AD (2011). Cognitive control and right ventrolateral prefrontal cortex: reflexive reorienting, motor inhibition, and action updating. *Ann N Y Acad Sci* **1224**: 40–62.

Litt A, Eliasmith C, Thagard P (2008). Neural affective decision theory: Choices, brains, and emotions. *Cogn Syst Res* **9**: 252–273.

Maldjian JA, Laurienti PJ, Kraft RA, Burdette JH (2003). An automated method for neuroanatomic and cytoarchitectonic atlas-based interrogation of fMRI data sets. *Neuroimage* **19**: 1233–1239.

McLaren DG, Ries ML, Xu G, Johnson SC (2012). A generalized form of context-dependent psychophysiological interactions (gPPI): A comparison to standard approaches. *NeuroImage* **61**: 1277–1286.

Murty VP, Shermohammed M, Smith DV, Carter RM, Huettel SA, Adcock RA (2014). Resting State Distinguish Human Ventral Tegmental Area from Substantia Nigra. *NeuroImage* **100**: 580–589.

Oldfield RC (1971). The assessment and analysis of handedness: the Edinburgh inventory. *Neuropsychologia* **9**: 97–113.

Patton JH, Stanford MS, Barratt ES (1995). Factor structure of the Barratt impulsiveness scale. *J Clin Psychol* **51**: 768–774.

Pedroni A, Koeneke S, Velickaite A, Jäncke L (2011). Differential magnitude coding of gains and omitted rewards in the ventral striatum. *Brain Res* **1411**: 76–86.

Petry J (Beltz, Psychologie-Verlag-Union: 1996). *Psychotherapie der Glücksspielsucht*. .

Petry J, Baulig T (1996). Kurzfragebogen zum Glücksspielverhalten. *Psychother Glücksspielsucht Psychol Verl Union* **97**: .

Sokol-Hessner P, Camerer CF, Phelps EA (2013). Emotion regulation reduces loss aversion and decreases amygdala responses to losses. *Soc Cogn Affect Neurosci* **8**: 341–350.

Sutton RS (MIT Press: Cambridge, Mass, 1998). *Introduction to reinforcement learning*. .

Tom SM, Fox CR, Trepel C, Poldrack RA (2007). The Neural Basis of Loss Aversion in Decision-Making Under Risk. *Science* **315**: 515–518.

Tzourio-Mazoyer N, Landeau B, Papathanassiou D, Crivello F, Etard O, Delcroix N, *et al* (2002). Automated Anatomical Labeling of Activations in SPM Using a Macroscopic Anatomical Parcellation of the MNI MRI Single-Subject Brain. *NeuroImage* **15**: 273–289.

Venkatraman V, Huettel SA, Chuah LYM, Payne JW, Chee MWL (2011). Sleep Deprivation Biases the Neural Mechanisms Underlying Economic Preferences. *J Neurosci* **31**: 3712–3718.

Wechsler D (The Psychological Corporation: San Antonio, TX, 1997). *Wechsler Adult Intelligence Scale - Third Edition*. .

Yang X, Beason-Held L, Resnick SM, Landman BA (2011). Biological parametric mapping with robust and non-parametric statistics. *NeuroImage* **57**: 423–430.
